# Responsible models and indicators: challenges from artificial intelligence

**DOI:** 10.3389/frma.2023.1305692

**Published:** 2023-10-18

**Authors:** Yi Zhang

**Affiliations:** Australian Artificial Intelligence Institute, Faculty of Engineering and Information Technology, University of Technology Sydney, Sydney, NSW, Australia

**Keywords:** responsibility, reliability, reproducibility, explainability, transparency, inclusiveness

## Introduction

Acknowledging the leadership of Wagner (2020) and her insightful comments on the three critical challenges to the community of *Research Policy and Strategic Management*, i.e., openness, relevance, and trust, our section has made remarkable strides over the past 3 years, attracting more than 100 high-quality articles and delving into a wide spectrum of topics within science, technology, and innovation policy, e.g., open science, science diplomacy, research collaboration, and sustainable development goals (SDG).

In the context of the global pandemic, it is noteworthy to observe the rapid response of the entire scientific community, manifesting in a significant upsurge in scientific publications. Meanwhile, the transformative advancements in artificial intelligence (AI), especially its horizontal applications in diverse sectors, such as ChatGPT, have been reshaping the cognitive processes and analytical paradigms of scientific research. *Frontiers in Research Metrics and Analytics* has actively embraced AI, e.g., machine learning-based classifiers and measures (Singhal et al., 2021; Mohlala and Bankole, 2022), embedding-based bibliometric studies (He and Chen, 2018; Wu et al., 2021), graph representation learning (Asada et al., 2021), and extensive utilization of academic graphs (Porter et al., 2020; Negro et al., 2023). Notebly, the community of *Research Policy and Strategic Management* has been actively engaged in in-depth discussions on trendy topics, such as responsible research and innovation (Buchmann et al., 2023), governance with equality and abundance (Kop, 2022), open data (Porter and Hook, 2022), and AI governance (Kalenzi, 2022).

It is imperative to acknowledge that while AI brings substantial benefits in terms of effective data analytics and knowledge discovery, we must remain vigilant about the challenges it presents to our community—a sort of Pandora's Box of AI challenges. Given the emphasis on the responsible development and utilization of AI-empowered models and indicators in the expansive domain of research policy and strategic management, we underscore three fundamental challenges: reliability and reproducibility, explainability and transparency, and inclusiveness.

## Challenge 1: reliability and reproducibility

Reliability and reproducibility stand as fundamental principles, emphasizing the importance of establishing a robust theoretical foundation and validating methodologies through rigorous experiments. Essentially, this entails a clear articulation of what we propose to utilize and why, and whether readers and peers can replicate our methods.

While our community may not be at the forefront of developing cutting-edge AI models, there is a growing trend among us to extensively utilize existing AI models for the analysis of science, technology, and innovation policy, e.g., AI-empowered variables and indicators for measurements, clustering, classification, and prediction. We welcome and value this cross-disciplinary interaction. However, a key challenge lies in substantiating the reliability of these AI models through compelling arguments, theoretical underpinnings, and empirical validation.

Reviewers frequently inquire the rationale behind the choice of a specific model over others, whether the model is customized or adaptable to a broad range of cases, and the strategy behind parameter settings, including its potential impact on robustness. We have observed that the most advanced models may not necessarily be the most suitable for our specific case, and the superficial use of AI without a comprehensive understanding of its underlying mechanisms can pose risks.

Our community has a strong inclination toward a hybrid approach combining quantitative and qualitative methodologies. We acknowledge the incredible success of this approach in balancing the objectivity of data analytics with the subjectivity of human knowledge, as well as addressing issues related to data bias and professional expertise. However, another critical challenge for scientific studies is the reproducibility of results. This aspect can be significantly influenced by factors such as the selection of expert panels, the presentation and visualization of results, and variations in interpretations of AI. Therefore, beyond the algorithms and parameters of AI models, we strongly advocate for a meticulous examination of reproducibility concerns throughout the entire academic research, commencing right from the methodological design phase. It is our collective responsibility to report all sensitive factors, accompanied by insights gleaned from the case study.

## Challenge 2: explainability and transparency

The principles of explainability and transparency represent advanced concepts that highlight the importance of interpreting the entire analytical process and identifying the influential factors that shape this process. In essence, this challenge revolves around comprehending the step-by-step generation of results through our proposed/chosen methods.

A central focus of our community is to inform science and public policy through both quantitative and qualitative evidence. Given the increasing involvement of AI in our work, it has become vital to provide comprehensive and convincing explanations for the results generated by AI models. This aids policymakers in addressing the “why” and “how” questions, enabling them to make informed decisions. The AI community has been actively advancing the field of explainable AI, equipping AI models with the capabilities to elucidate their decisions, recommendations, predictions, and the process underlying these actions (Gunning et al., 2019). We have observed numerous applications of explainable AI in information studies, e.g., identifying the most influential team features in determining performance. This presents our community with significant opportunities to explore its feasibility.

Expanding the concept of explainability, we embrace a broader perspective under the term “transparency.” This emphasizes the challenge facing all decision support approaches in the realm of science, technology, and innovation policy. Whether it is a sophisticated AI model with explainable functionalities or a qualitative study reliant on workshops and expert engagement, we expect a comprehensive account of the entire research progress in research articles. This includes detailing the stepwise procedures employed in workshops for technology roadmaps, providing in-depth description of questionnaires used in surveys of interdisciplinary experts, and presenting the complete set of questions and answers from interviews conducted with entrepreneurs and policymakers.

## Challenge 3: inclusiveness

Inclusiveness aligns seamlessly with the contemporary societal pursuit of diversity, equity, inclusion, and belonging (DEIB), with a strong emphasis on addressing DEIS issues within academia and across a broad range of scientific events and activities, as well as the development and application of responsible indicators and models for underrepresented individuals, population groups, and communities.

Within the wide context of scientific topics, such as science of science, our community has undertaken commendable efforts in analyzing gender inequality (Jackson et al., 2022) and crafting inclusive metrics (Pourret et al., 2022). However, a significant practical challenge lies in scaling up these analyses from specific regions, individual disciplines, and singular data sources to comprehensive global studies with integrated knowledge graphs. Additionally, it involves providing empirical insights into DEIB while offering actionable political recommendations. Establishing an inclusive platform to study, comprehend, and foster DEIB within our journal, our scientific community, and society at large must be regarded as a long-term and substantial objective.

Among the trendy interests within the AI community, notable developments have emerged in responsible AI (Dignum, 2019), with a specific glance at inclusiveness, e.g., fair graph representation learning to address biases in demographical attributes (Subramonian et al., 2022). In light of these AI advancements, the challenge to our community is to construct a persuasive, feasible, and narrative-driven framework for integrating these innovative developments into our empirical studies.

## Conclusions

In the current landscape, where the imperative for responsible AI, ethical AI, and trustworthy AI extends beyond the AI community to encompass society at large, our community's dynamic engagement with AI necessitates a deliberate and strategic approach to transform this challenge into significant opportunities. In conjunction with the challenges of reliability and reproducibility, explainability and transparency, and inclusiveness, we strongly advocate for the community of *Research Policy and Strategic Management*, as well as the *Frontiers in Research Metrics and Analytics* community at large, to proactively address this AI-driven paradigm shift. It is essential for us to embrace our leadership roles and take pre-emptive actions that not only respond to but also guide this revolution age. By doing so, we can fulfill our commitment to informing global science and public policy effectively and responsibly.

## Author contributions

YZ: Writing—original draft, Writing—review and editing.
